# Pyrosequencing reveals diverse fecal microbiota in Simmental calves during early development

**DOI:** 10.3389/fmicb.2014.00622

**Published:** 2014-11-17

**Authors:** Daniela Klein-Jöbstl, Elisa Schornsteiner, Evelyne Mann, Martin Wagner, Marc Drillich, Stephan Schmitz-Esser

**Affiliations:** ^1^Clinical Unit for Herd Health Management, Department for Farm Animals and Veterinary Public Health, University Clinic for Ruminants, University of Veterinary Medicine ViennaVienna, Austria; ^2^Research Cluster “Animal Gut Health,” University of Veterinary Medicine ViennaVienna, Austria; ^3^Department for Farm Animals and Veterinary Public Health, Institute for Milk Hygiene, Milk Technology and Food Science, University of Veterinary Medicine ViennaVienna, Austria

**Keywords:** fecal bacterial community, calf, 16S rRNA amplicon pyrosequencing, Simmental breed, early development

## Abstract

From birth to the time after weaning the gastrointestinal microbiota of calves must develop into a stable, autochthonous community accompanied by pivotal changes of anatomy and physiology of the gastrointestinal tract. The aim of this pilot study was to examine the fecal microbiota of six Simmental dairy calves to investigate time-dependent dynamics of the microbial community. Calves were followed up from birth until after weaning according to characteristic timepoints during physiological development of the gastrointestinal tract. Pyrosequencing of 16S rRNA gene amplicons from 35 samples yielded 253,528 reads clustering into 5410 operational taxonomic units based on 0.03 16S rRNA distance. Operational taxonomic units were assigned to 296 genera and 17 phyla with *Bacteroidetes*, *Firmicutes*, and *Proteobacteria* being most abundant. An age-dependent increasing diversity and species richness was observed. Highest similarities between fecal microbial communities were found around weaning compared with timepoints from birth to the middle of the milk feeding period. Principal coordinate analysis revealed a high variance particularly in samples taken at the middle of the milk feeding period (at the age of approximately 40 days) compared to earlier timepoints, confirming a unique individual development of the fecal microbiota of each calf. This study provides first deep insights into the composition of the fecal microbiota of Simmental dairy calves and might be a basis for future more detailed studies.

## Introduction

Cattle nutrition changes fundamentally from birth to adulthood. Cattle are typical foregut fermenters undergoing considerable changes in anatomy and physiology of the gastrointestinal tract during the first weeks of life. From a functional point of view, the newborn calf is a non-ruminant. In the first 2–3 weeks of life the calf relies almost entirely on milk or milk replacer and consumes negligible amounts of solid feed. After this period, the calf starts consuming increasing amounts of roughage and concentrates enhancing the development of the forestomach, and finally evolving all functional properties of a ruminant (Drackley, 2008). These changes of the gastrointestinal tract are accompanied by maturing processes of the gastrointestinal microbial community, initiated by the rapid colonization of the intestinal tract post natum. It has been demonstrated that diet has a substantial effect on the composition of the gastrointestinal microbiota (Callaway et al., 2010; Maslowski and Mackay, 2011). In addition, recent studies have shown that the calf feeding-management (type and amount of feed offered, feeding techniques) and early forestomach development depending on solid feed-intake positively influences overall gut health and productivity (Cozzi et al., 2002; Heinrichs and Heinrichs, 2011; Soberon et al., 2012). It is however, widely unknown how these factors contribute to shifts in the gastrointestinal microbiota. So far, mainly cultivation-based examinations of selected bacteria, particularly pathogens have been performed, identifying only a small fraction of the calves' intestinal microbiota (Nocker et al., 2007). Cultivation-independent techniques allow for the examination of the composition of the whole bacterial microbiota. Particularly high-throughput sequencing technologies, such as Roche/454 pyrosequencing or Illumina sequencing technology are now widely used for the characterization of microbial communities by sequencing 16S rRNA gene PCR amplicons, providing unprecedented sampling depth (Kuczynski et al., 2011, 2012). Recent studies analyzing cattle microbiota almost exclusively focused on the Holstein-Friesian breed (Dowd et al., 2008; Patton et al., 2009; McGarvey et al., 2010; Uyeno et al., 2010; Oikonomou et al., 2013; Malmuthuge et al., 2014). In the present study, calves of the Simmental breed, the predominant dairy breed in the European Alpine regions, were chosen. In contrast to Holstein-Friesian, Simmental is a typical double purpose breed (beef and milk), differing in feed, nutrient, and trace element intake as well as in metabolic body weight compared with other breeds (Jenkins and Ferrell, 2007; Fry et al., 2013; Gruber et al., 2014). Calves in the Alpine region are mainly fed with whole milk and not with milk replacer as Holstein-Friesian calves in other studies (Uyeno et al., 2010; Edrington et al., 2012; Oikonomou et al., 2013; Klein-Jöbstl et al., 2014; Stanìk et al., 2014). Milk replacer may be of reduced energy density compared with whole milk (Godden et al., 2005; Moore et al., 2009). Simmental calves of the Alpine region are generally weaned at the age of approximately 10–11 weeks (Klein-Jöbstl et al., 2014), whereas in other studies using Holstein-Friesian breed weaning was often enforced earlier (Oikonomou et al., 2013). Furthermore, calves have access to hay early in life, which is in contrast to most published studies examining the microbiota of Holstein-Friesian calves (Edrington et al., 2012; Oikonomou et al., 2013). In agreement with studies dealing with gastrointestinal microbiota in calves (Li et al., 2012; Jami et al., 2013), we believe that monitoring gut microbiota under standard weaning conditions is important because the composition of the gut microbiota in calves in the weaning period is still largely unknown, particularly for calves of breeds other than Holstein-Friesian. A better understanding of the development of the gut microbiota community composition is even more important in light of the major changes in physiological development of calves during this time of their lives.

The objectives of this pilot study were: (i) to provide first insights into the fecal bacterial microbiota of Simmental calves to examine for the communities acquired after birth and the changes in these bacterial communities at different stages of the animals' development and (ii) to compare our results with studies using feces from Holstein-Friesian cattle. Our study aims to provide a basis for further studies focusing on calf-microbe interactions in early development or examining factors that influence the gastrointestinal microbiota in calves.

## Materials and methods

### Animals and sampling

This study was discussed and approved by the institutional ethics committee of the University of Veterinary Medicine Vienna in accordance with Good Scientific Practice and national legislation.

Six Simmental calves, born and reared on the Teaching and Research Farm of the University of Veterinary Medicine Vienna, were included in this study. Calves were born in a group calving pen between February and May 2012; all calves were delivered vaginally. Calves were separated from their dam immediately after birth. Each calf received 4 l of colostrum from its own mother fed by a bucket within 6 h post natum. No routine treatments were performed except navel disinfection. Calves were housed in single calf hutches outside the stable for approximately 2 weeks. Afterwards, calves of similar ages were grouped and housed in an outdoor climate stable. During the first week of life calves were fed with sellable pasteurized whole milk three times a day, afterwards twice a day with the total amount of milk according to 12% of the calves' body weight. No waste milk (milk from cows treated with antibiotics or from cows with clinical mastitis) was fed to the animals. Hay was offered *ad-libitum* from the second day of life. Concentrates were fed from the start of group housing. Calves were weaned at an age of approximately 11 weeks (mean 78 days, with a range from 65 to 94 days).

Fecal samples were taken six times per calf. Sampling timepoints were based on the standard feeding management on farm (Supplementary Figure 1). The first sample was taken within 12 h after birth, the second in the second week (day 8–14) of life, the third in the week after addition of concentrates to the diet (week 3 of life), the fourth sample was taken in the middle of the milk feeding period (5th–6th week of life) and the last two sampling times were in the week before and after weaning. Fecal samples were taken directly from the rectum by use of sterile gloves and lubricant. Feces were put into sterile plastic tubes and either frozen at −20°C or processed immediately.

### DNA isolation, preparation of 16S rRNA gene amplicon libraries and pyrosequencing

Genomic DNA was isolated from 200 mg feces using the QIAamp DNA Stool Mini Kit (Qiagen, Hilden, Germany) according to the manufacturers' guidelines with the protocol for Gram-positive bacteria. DNA concentration was determined by a Qubit fluorometer (Invitrogen, Vienna, Austria).

16S rRNA genes were amplified using FLX 454 one way read barcoded fusion primers with the template specific primer sequence GM3 (5′- AGAGTTTGATCMTGGC 3′) and 926R (5′- CCGTCAATTCMTTTGAGTTT 3′) (Muyzer et al., 1995) targeting the V1–V4 hypervariable regions of the 16S rRNA gene. For each sample, a PCR mix of 20.0 μl was prepared containing: 4.0 μl BioStab PCR Enhancer (Sigma-Aldrich, Vienna, Austria), 4.0 μl buffer A, 0.6 μl MgCl_2_ (25 mM), 0.6 μl dNTP Mix, 0.2 μl Kapa2G-Robust Polymerase (Kapa Biosystems, Wilmington, MA, USA), 1.0 μl tagged GM3 primer, and tagged 926R primer (each 10 pmol/μl), 1.0 μl template, 1.0 μl Tth-recA (100 ng/μl, McLab, San Francisco, USA), 1.0 μl ATP (20 mM). Thermal cycling conditions on a SensoQuest LabCycler (SensoQuest, Göttingen, Germany) were: initial denaturation at 96°C for 2 min followed by 35 cycles of denaturation at 96°C for 15 s, annealing at 50°C for 15 s and extension at 72°C for 1 min with a final extension of 7 min at 72°C. Amplicons were purified using the Qiagen MinElute kit (Qiagen, Hilden, Germany). Sequencing of an equimolar pool of 35 samples on 1/2 PicoTiterPlate was performed using the GS FLX+ Titanium Sequencing Kit XLR70 (Roche 454 Life Science, Branford, CT, USA) according to manufacturers' instructions. PCR amplification, library preparation, emulsion PCR, and sequencing was performed by LGC Genomics (Berlin, Germany).

### Data analysis

All reads were processed with mothur (Schloss et al., 2009) according to the procedure described by Schloss et al. (2011). Briefly, primer, barcode sequences, and sequences of low quality and length were trimmed with the following parameters: Minimum average quality score (using a window size of 50 bp): 35; minimum read length: 290 bp; maximum number of differences to primer sequence: two, ambiguous bases were not allowed; maximal homopolymer length: eight; and maximum number of differences to the barcode: one. Pyrosequencing error was reduced using the “pre.cluster” algorithm, chimeric sequences were excluded with “chimera.uchime.” The remaining high quality reads were assigned to a reference taxonomy, the SILVA reference database (Pruesse et al., 2007), using the RDP naïve Bayesian rRNA classifier (confidence threshold = 80%). Sequences were clustered into operational taxonomic units (OTU) using a distance limit of 0.03 (97% similarity). This resulting classification was used for all further downstream analyses on all taxonomic levels. For calculation of diversity indices, the number of reads in all samples was normalized to 1500 sequences using the “sub.sample” option in mothur. Species richness and diversity indices were estimated by calculating ACE and Chao1 as well as the Shannon and Simpson diversity indices using mothur. The comparative results of species richness and diversity indices were visualized as box-and-whisker plots showing the median and the interquartile (midspread) range (boxes containing 50% of all values), the whiskers (representing the 25 and 75 percentiles) and the extreme data points. Differences in richness and diversity indices as well as in abundance of genera between timepoints were examined by paired Wilcoxon test with Bonferroni correction using PASW, version 20.0 (IBM Cooperation, New York, USA). Rarefaction curves were generated using mothur and Sigmaplot 11.0 (Systat Software, Chicago, USA). Comparison of community structure between sampling timepoints was performed with Libshuff incorporated in mothur. Differences were considered significant at *P* < 0.05.

Heatmaps were created using JColorGrid (Joachimiak et al., 2006). To assess how distinct the fecal microbiota of each timepoint was from each other, we compared the level of similarity of bacterial community structures using the Morisita–Horn similarity index calculated with mothur.

Principal coordinates analysis (PCoA) was performed using the sampling timepoints as variables and prevalence of the 18 most abundant genera as covariates. These 18 genera include 96% of all sequences. The PCoA plot and a component loading plot with each vector corresponding to one genus being proportional to its component loading were calculated and visualized in JMP Pro (SAS Institute).

The pyrosequencing data are available in the EMBL SRA database under the accession number PRJEB4554.

## Results

Pyrosequencing of the fecal samples yielded 846,902 reads with read lengths from 21 to 1087 bp. After rigorous quality control, 253,528 (29.9%) reads ranging from 290 to 600 bp remained for further analyses. On average, 7243 reads were obtained per sample (range: 827–21,949). For all further analyses reads were classified into 5410 operational taxonomic units (OTUs) using 0.03 16S rRNA distance. Of the 5410 OTUs, 3201 OTUs were represented only by a single read. The OTUs belonged to 296 genera and affiliated to 17 phyla.

**Figure 1:**
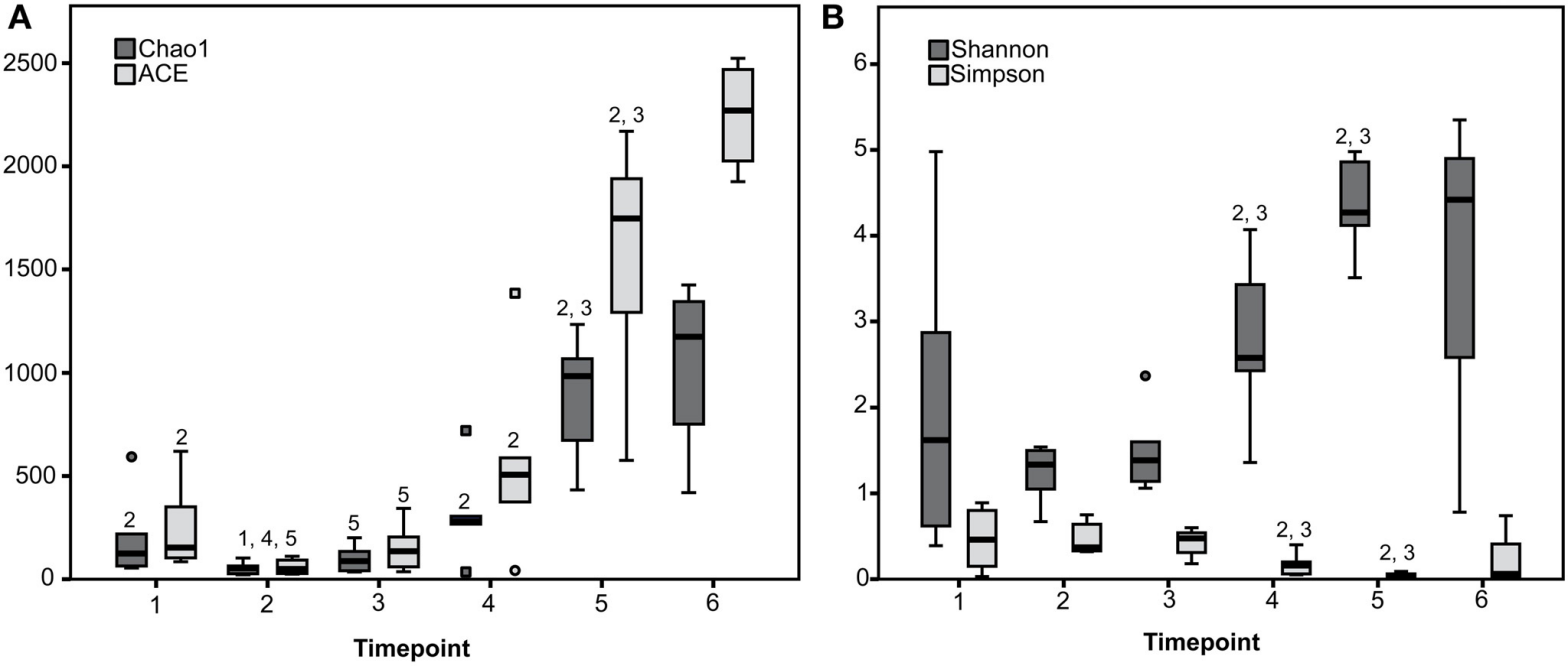
**Species richness estimates (Chao1 and ACE) (A) and diversity indices (Shannon and Simpson) (B) for the six sampling timepoints are presented**. ^1 to 6^ Estimate or index differs (*P* < 0.05) with the estimate or index of the timepoint with the given number (1 to 6).

Rarefaction curves revealed that diversity coverage was high for most samples (Supplementary Figure 2). Species richness and diversity significantly differed between timepoints showing a trend toward increasing diversity with advanced age and solid feed consumption (Figure 1).

In the following, data are presented analyzing all samples of each timepoint together using median values.

**Figure 2:**
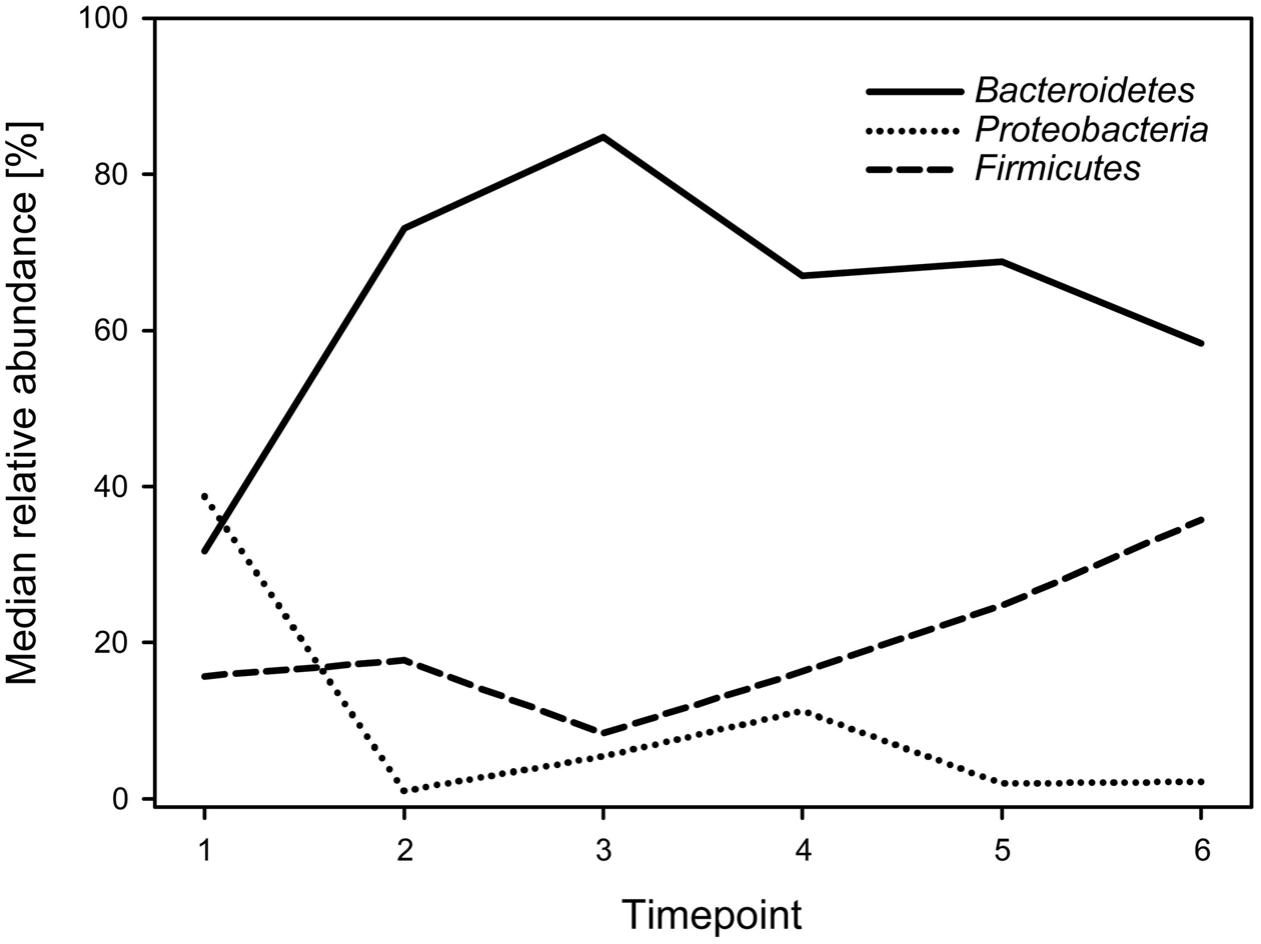
**Median relative abundances of the three most abundant phyla during the six timepoints**.

Regarding phyla, the calf fecal communities were dominated by *Bacteroidetes* (69.3%), *Proteobacteria* (15.7%), and *Firmicutes* (14.8%), accounting for 99.8% of all reads. *Bacteroidetes* dominated during all timepoints (with medians ranging from 31.7 to 84.8%) except for timepoint 1, when *Proteobacteria* showed the highest abundance with a median of 38.7%. While *Bacteroidetes* decreased, *Firmicutes* increased over time (Figure 2). The overall *Firmicutes* to *Bacteroidetes* ratio was 0.2 varying between 0.1 and 0.4 during the different timepoints, with a tendency to increase with age.

**Figure 3:**
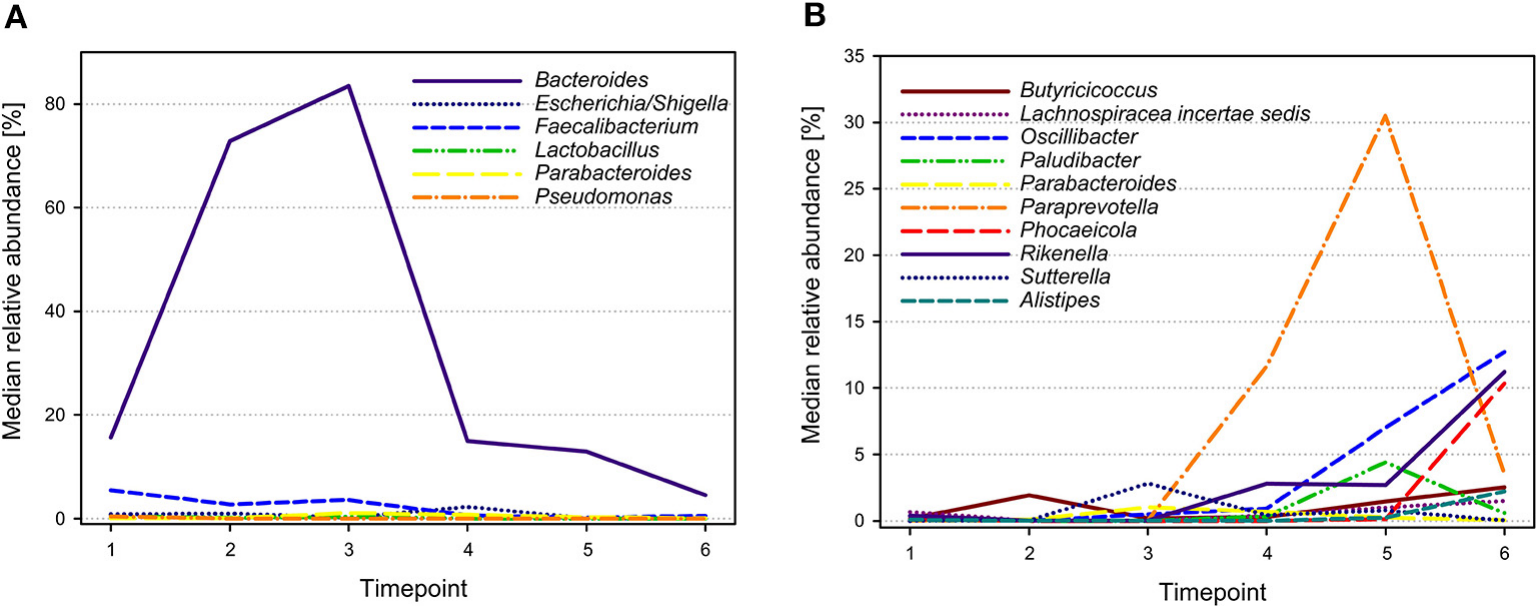
**Median relative abundances of selected genera during different timepoints**. Genera showing highest abundances during early timepoints of calf development are shown in **(A)**, whereas genera with highest abundances during later timepoints are shown in **(B)**.

The 28 most abundant genera (with a relative abundance of more than 0.1% among all samples) accounted for 95.9% of all reads (Table 1). The median prevalences of the most abundant genera by timepoint are presented in Figure 3. Numbers and significances are shown in Supplementary Table 1. Timepoints 1 to 3 are dominated by *Bacteroides*. The relative abundance of *Bacteroides* is generally decreasing over time but showed no statistically significant changes between the six timepoints. *Escherichia*-*Shigella* (with median relative abundances varying between 0.0 and 2.3%) was mainly associated with early timepoints 1 to 4. During timepoint 1 and 2, *Escherichia*-*Shigella* appeared significantly more often than at timepoints 5 and 6. *Lactobacillus* had relatively low abundances (overall relative abundance of 1.5%). Highest *Lactobacillus* abundances were found during the first three timepoints with a significant decrease over time. *Faecalibacterium* (overall relative abundance of 4.8%) also decreased significantly over time. In contrast, *Oscillibacter*, *Phocaeicola*, and *Alistipes* increased significantly over time. *Paraprevotella* increased from timepoint 1 to 5 and was most abundant during timepoint 5 (with a median of 30.5%). Although the median decreased afterwards, the relative abundance was significantly higher during timepoint 6 compared to timepoint 1.

**Table 1:** **Relative abundance of the 28 most abundant genera (relative abundance > 0.1%) among all samples**.

**Genus**	**No. of reads**	**Relative abundance (%)**
*Bacteroides*	144,517	57.00
*Escherichia*-*Shigella*	26,398	10.41
*Faecalibacterium*	12,097	4.77
*Paraprevotella*	10,969	4.33
*Sutterella*	7420	2.93
*Rikenella*	6666	2.63
*Butyricicoccus*	6519	2.57
*Parabacteroides*	4508	1.78
*Lactobacillus*	3683	1.45
*Oscillibacter*	3358	1.32
*Paludibacter*	2727	1.08
*Pseudomonas*	2615	1.03
*Phocaeicola*	2490	0.98
*Lachnospiracea incertae sedis*	2144	0.85
*Alistipes*	929	0.37
*Clostridium* XIVa	843	0.33
*Clostridium* XIVb	697	0.27
*Klebsiella*	641	0.25
*Flavonifractor*	585	0.23
*Sporobacter*	500	0.20
*Butyricimonas*	477	0.19
*Ahrensia*	457	0.18
*Odoribacter*	423	0.17
*Acinetobacter*	358	0.14
*Barnesiella*	302	0.12
*Ethanoligenens*	296	0.12
*Butyrivibrio*	293	0.12
*Clostridium* IV	283	0.11
Sum	243,195	95.92

In accordance with the overall low similarities, only 11 genera (representing 24 OTUs) are shared between all timepoints (data not shown). These taxa might represent the core microbiome of developing calves irrespective of the age. Most likely, these taxa play an important role in the fecal microbial communities. Using Libshuff we could show that the community structures of the different timepoints are different (*P* < 0.001, data not shown).

**Figure 4:**
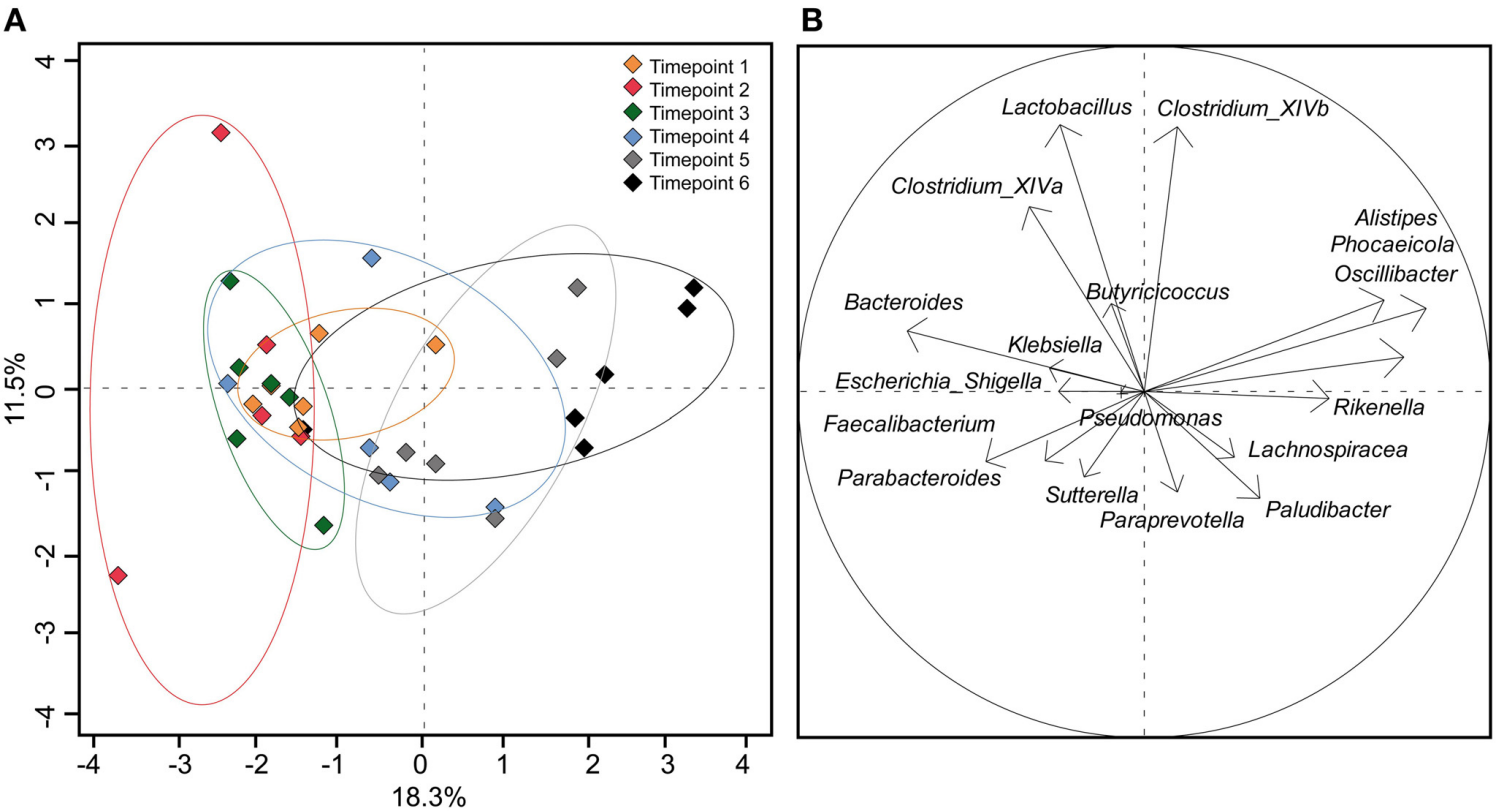
**(A)** PCoA plot depicting fecal samples of calves taken at different timepoints. **(B)** Component loading plot with each vector corresponding to one genus being proportional to its component loading. The percentage of variation explained (plotted principal coordinates) is indicated on the axes.

Using the 18 most abundant genera, PCoA revealed high similarity between timepoints 1, 2, and 3 (Figure 4). The discrimination and the high variance of timepoint 4 compared to earlier timepoints confirm the unique development of the microbial community of each calf around 40 days of age. Timepoint 5 was also compositionally distinct to timepoint 1–3 but was similar to timepoint 4 with less variance between calves' feces samples. Timepoint 6 revealed no clear distinction but showed low variance of discrimination between calves. This indicates that the calf fecal communities became more similar at the later timepoints, particularly after weaning.

One aim of our study was the comparison of our results from Simmental calves with Holstein-Friesian calves. For this, we joined our data with the data from Oikonomou et al. (2013) and created Venn diagrams to illustrate shared OTUs between the samples from different breeds: we found 614 (out of 10,289) OTUs to be shared between Holstein-Friesian and Simmental breeds (Supplementary Figure 3). Although the shared OTUs correspond to only 5.9% of all OTUs, these shared OTUs represent the most abundant OTUs and 83% of all sequences. This indicates that both breeds harbor a core microbiome consisting largely of abundant OTUs. Nevertheless, both breeds also contain a high number of distinct (mostly rare) OTUs.

## Discussion

The objective of the present study was to analyze the fecal bacterial community composition in Simmental calves from birth until after weaning and to identify changes in the fecal microbiota during normal development. In contrast to other studies that almost exclusively examined Holstein-Friesian calves, in this study Simmental calves were used, as this is the predominant dairy breed in the Alpine region (Klein-Jöbstl et al., 2014) and almost nothing is known about the microbiota of this breed. Studies examining different breeds in regard to feed and nutrient intake as well as digestibility of feed recognized breed differences (Jenkins and Ferrell, 2007; Fry et al., 2013; Gruber et al., 2014; Lourenço et al., 2014). Furthermore, not only the breed but also management and particularly feeding in our study differed from other studies (Uyeno et al., 2010; Edrington et al., 2012; Oikonomou et al., 2013). The management strategy of the animals in our study was typical for Austrian farms (Klein-Jöbstl et al., 2014), and included feeding with sellable whole milk, free access to roughage (hay) from the first week of life and supplying concentrates not before the third week of life. This is in contrast to studies conducted with Holstein-Friesian calves (Uyeno et al., 2010; Edrington et al., 2012; Oikonomou et al., 2013). Furthermore, in our study calves were weaned at the age of approximately 11 weeks, dependent of solid feed intake, whereas in the study by Oikonomou et al. (2013) weaning was enforced in the seventh week of the calves' life. Apart from differences in intake and digestibility of feed between breeds it is obvious that differences between results of studies with Holstein-Friesian and Simmental calves cannot clearly be attributed to breed. These findings could be interpreted as differences between calf rearing systems including the aforementioned factors and could be considered for developing strategies to improve calves gut health. This could be of interest for feeding or treatment strategies. In this context, knowledge of the diversity of the intestinal microbiota and changes during the rearing period are essential.

It is known that the microbiota of the digesta differs from the gastrointestinal mucosa microbiota (Malmuthuge et al., 2012, 2014). The study of Malmuthuge et al. (2014) revealed similar bacterial communities when comparing mucosa and digesta of the large intestine, whereas in the small intestine differences were more distinct. In the present study, we choose to examine feces as this is a non-invasive, practicable and widely used method to sample animals repeatedly (Callaway et al., 2010; McGarvey et al., 2010; Mao et al., 2012; Oikonomou et al., 2013; Kim et al., 2014).

Although all calves belonged to the same breed and were reared on the same farm under the same management conditions, the combination of the microbial communities acquired and maintained was distinct between most of the calves, which is in accordance with findings in humans during early development (Palmer et al., 2007). Nevertheless, some significant changes and tendencies could be determined: Species richness and diversity increased over time. Highest species richness and diversity was detected in the weeks before and after weaning (timepoints 5 and 6) when calves consumed largest amounts of solid feed. In contrast, during early timepoints the fecal bacterial microbiota was often dominated by one genus (*Bacteroides*).

An increase in species richness and diversity has been observed in response to different feeding (Malmuthuge et al., 2013) or increasing age (Edrington et al., 2012; Oikonomou et al., 2013) in calves, as well as in children (Fan et al., 2013). Furthermore, in our study later timepoints (5 and 6) became more similar to each other as shown in the PCoA plot, indicating that the fecal microbial communities stabilize around weaning.

In accordance with studies from human and animal feces few phyla (*Bacteroidetes*, *Firmicutes*, and *Proteobacteria*) were present in all samples and accounted for most reads (Lee et al., 2011; Shanks et al., 2011; Malmuthuge et al., 2014). In contrast to most studies in adult cattle, in which *Firmicutes* dominated (Ozutsumi et al., 2005; Durso et al., 2011; Shanks et al., 2011; Edrington et al., 2012; Mao et al., 2012; Kim et al., 2014), in our study *Bacteroidetes* showed the highest overall relative abundance with 69.3% and dominated in most samples. Despite the differences between the human and ruminant gastrointestinal tract, our findings are consistent with studies examining feces of newborn babies (Mariat et al., 2009). Furthermore, in the study of Malmuthuge et al. (2014) tissue as well as digesta samples of the large intestine of pre-weaned calves were also dominated by *Bacteriodetes*. The authors assumed that *Bacteroidetes* tend to more readily colonize the large intestine of calves. The overall *Firmicutes* to *Bacteroidetes* ratio is regarded to be of relevance in the human gut microbiota composition, especially in connection with energy harvesting (Ley et al., 2006). This ratio showed a mean of 0.2 in the calves in our study and is similar to that of infants (Mariat et al., 2009). In contrast, Oikonomou et al. (2013) detected a significantly higher relative abundance of *Firmicutes* (63.8–81.9%) in Holstein-Friesian calves, resulting in *Firmicutes* to *Bacteroidetes* ratios varying from 6.2–46.1, which are similar to ratios in adult cattle.

The high relative abundance of *Bacteroidetes* in our study can largely be explained by OTUs belonging to the genus *Bacteroides*, which was particularly abundant in calves before weaning (timepoints 1–4), and is in accordance with findings in the rumen of developing calves (Li et al., 2012; Wu et al., 2012; Jami et al., 2013). It has been shown that *Bacteroides* was, among others, present in the vagina of healthy cows (Santos et al., 2011). Studies in humans have shown that the first fecal bacterial communities found in vaginally born newborns depend on the vaginal microbiota (Dominguez-Bello et al., 2010). Thus, the high relative abundance of *Bacteroides* in our calves, particularly at early timepoints, could have also been influenced by ingestion during passage through the birth canal. In adult cattle, the relative abundance of *Bacteroides* in the feces was negatively associated with a high fiber diet (Kim et al., 2014). Consequently, increased fiber ingestion and decreased milk consumption during the calves' development might be a reason for the decrease in *Bacteroides* over time. *Escherichia*-*Shigella* was almost exclusively detected during the first four sampling timepoints similar to the findings of Edrington et al. (2012).

Similarly, *Lactobacillus* was predominantly found during the first four sampling timepoints, although at a relatively low abundance. The early appearance of lactobacilli might be due to milk feeding or the incorporation of these bacteria during passage of the vagina during natural birth, as shown in humans (Palmer et al., 2007; Dominguez-Bello et al., 2010). A decrease of lactobacilli over time was furthermore reported with reduction of milk feeding and weaning, respectively (Vlková et al., 2006; Uyeno et al., 2010; Oikonomou et al., 2013). Similarly, *Faecalibacterium* reached highest abundances within the first three sampling times.

In contrast, around weaning the relative abundance of *Paraprevotella*, *Oscillibacter*, *Alistipes*, and *Phocaeicola* increased. These bacteria have also been found in notable abundance in the rumen and feces of adult cattle and are known to contribute to basic functions of the rumen ecosystem (Pitta et al., 2010; Durso et al., 2012; Wu et al., 2012; Kim et al., 2014). The relative abundance of bacteria of the *Prevotella* group (including *Paraprevotella*) as well as of *Oscillibacter* are influenced by a high fiber diet (De Filippo et al., 2010; Walker et al., 2011; Wu et al., 2011; Rice et al., 2012; David et al., 2014). Similar to the findings in our study, *Oscillibacter*, a butyrate producer, increased in the rumen of preweaned calves with age (Li et al., 2012). In conclusion, the proliferation of the aforementioned bacteria in the calves of our study may be explained by decreasing milk availability around weaning and a concomitant increase in the diet fiber content by increasing hay consumption.

One highly abundant group of bacteria particularly in breast-fed infants are bifidobacteria. We did not detect bifidobacteria in our study. One reason for the presumptive absence of bifidobacteria might be that the reverse primer used in our study shows one mismatch to the genus *Bifidobacterium* (Sim et al., 2012). However, a number of recent studies also reported the absence or only low abundance of bifidobacteria in calves and cattle (Vlková et al., 2008; Uyeno et al., 2010; Lee et al., 2011; Kim et al., 2014; Malmuthuge et al., 2014). Collectively, these studies suggest that the presence of bifidobacteria is dependent on the feed: a combined diet of milk and hay (as fed in our study) leads to a significant reduction of bifidobacteria (Vlková et al., 2008).

Our results showed a pronounced shift in the fecal bacterial composition from the beginning of life, when calves predominantly consume milk, to later timepoints, when calves ingest increasing amounts of solid feed and are weaned. Similar changes in the ruminal microbiota have been described in previous studies. It can be assumed that during early development these changes go hand in hand, whereas during later timepoints, when the gastrointestinal tract stabilizes, the microbiota of the fore- and hindgut differentiate.

Although the number of animals examined in our study are within the range of other studies (Callaway et al., 2010; Godoy-Vitorino et al., 2012; Li et al., 2012; Jami et al., 2013; Kim et al., 2014), the relatively low number of examined calves has to be taken into account when interpreting the results. To detect at least minimal effects, the sample size would have to be 28 animals what was due to financial reasons not possible in this pilot study aiming to get first insights into the microbiota of Simmental calves.

In conclusion, in this study we present first insights into the fecal microbial community of Simmental calves. Further studies will be needed to examine factors that may influence the fecal bacterial microbiota like disease, especially calf diarrhea, different feeds or feeding managements, feed additives or antibiotic treatments.

Although the fecal microbial communities in calves showed inter-individual variation, we detected an increase in diversity and species richness of the fecal bacterial community in calves. Concurrently, an increasing within-age-group similarity and a shift in the bacterial composition from early timepoints (from birth to the middle of the milk feeding period) to the timepoints around weaning could be observed. In order to elucidate differences and similarities in the microbial communities in cattle of different breed, age, management, or geographic origin, more standardized studies are needed in order to improve the comparability among different studies. This is also relevant for amplicon sequencing studies in which a more standardized methodology (e.g., using the same DNA isolation protocols, same PCR primers) is needed in the future.

The gastrointestinal microbiota has a significant impact on health and productivity of animals as well as on disease, therefore further studies are necessary to unravel the function of specific microbes during early calf development.

### Conflict of interest statement

The authors declare that the research was conducted in the absence of any commercial or financial relationships that could be construed as a potential conflict of interest.
