# Supplementary material for: Pyrosequencing reveals diverse fecal microbiota in Simmental calves during early development
Source: Front Microbiol. 2014 Nov 17;5:622. doi: 10.3389/fmicb.2014.00622 (PMC4233928; doi:10.3389/fmicb.2014.00622)
Supplement: Supplementary file 1 [file Presentation1.PDF]

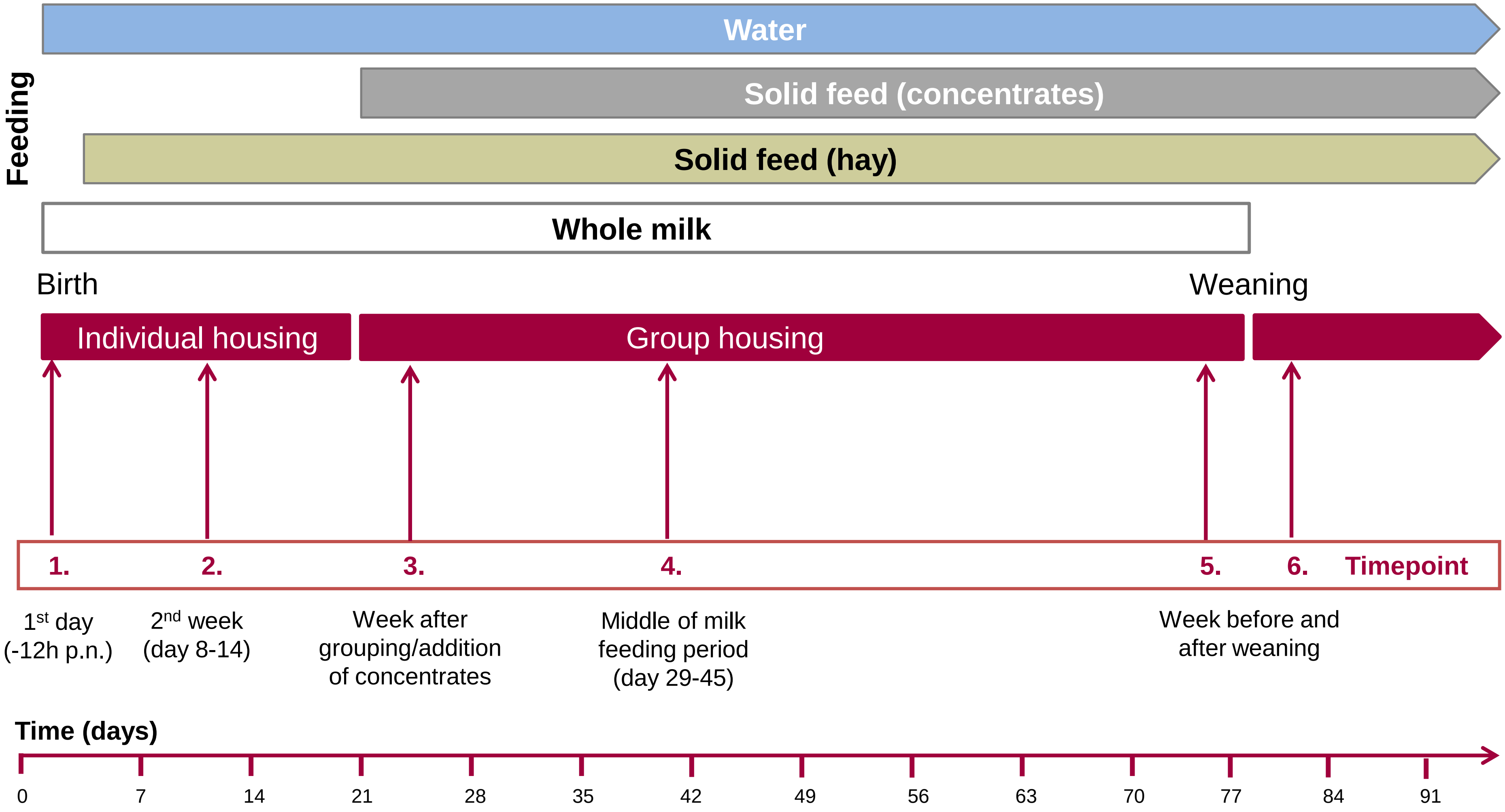

Supplementary Figure 1. Illustration of routine calf feeding and housing on the study farm and presentation of the six sampling timepoints
