# Supplementary material for: Pyrosequencing reveals diverse fecal microbiota in Simmental calves during early development
Source: Front Microbiol. 2014 Nov 17;5:622. doi: 10.3389/fmicb.2014.00622 (PMC4233928; doi:10.3389/fmicb.2014.00622)
Supplement: Supplementary file 2 [file Presentation2.PDF]

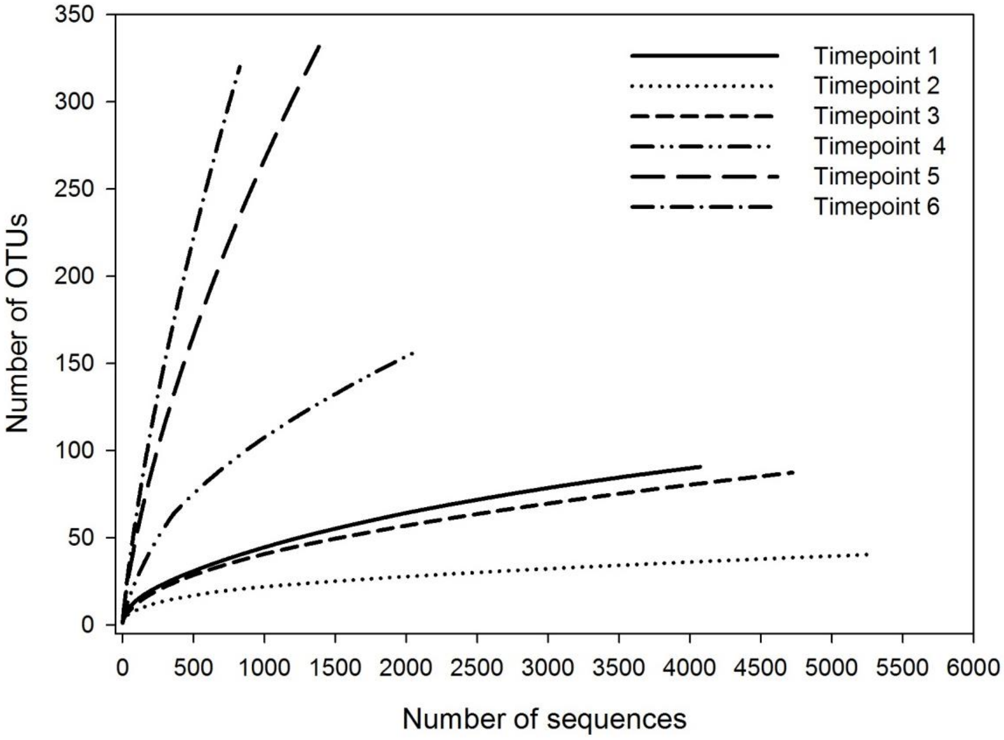

Supplementary Figure 2. Rarefaction analyses for each timepoint. Within each timepoint, median values were calculated and are depicted.
