# Supplementary material for: Pyrosequencing reveals diverse fecal microbiota in Simmental calves during early development
Source: Front Microbiol. 2014 Nov 17;5:622. doi: 10.3389/fmicb.2014.00622 (PMC4233928; doi:10.3389/fmicb.2014.00622)
Supplement: Supplementary file 3 [file Presentation3.PDF]

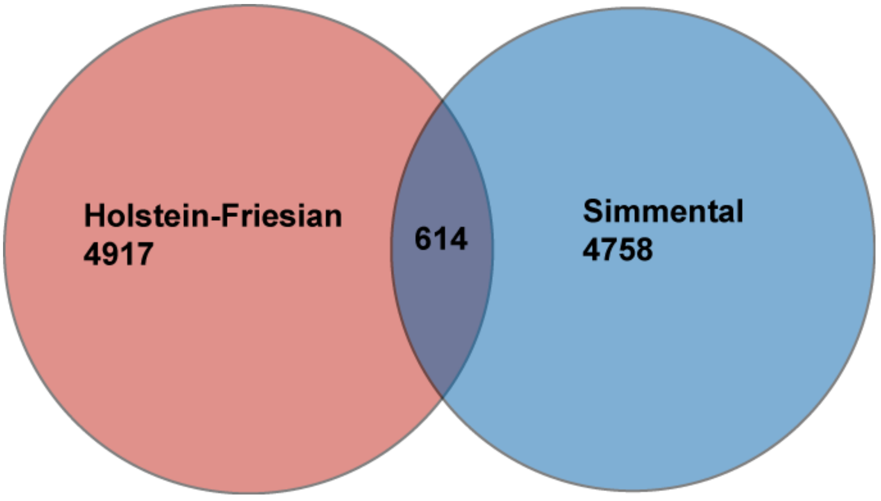

Supplementary Figure 3: Venn Diagram showing shared OTUs between fecal samples from Holstein-Friesian and Simmental-breed calves. The number of OTUs (based on 0.03 16S rRNA gene distance) are indicated. The data from Holstein-Friesian calves were taken from Oikonomou et al. (2013 *PLoSOne*. 8, e63157).
