# Supplementary material for: Pyrosequencing reveals diverse fecal microbiota in Simmental calves during early development
Source: Front Microbiol. 2014 Nov 17;5:622. doi: 10.3389/fmicb.2014.00622 (PMC4233928; doi:10.3389/fmicb.2014.00622)
Supplement: Supplementary file 4 [file DataSheet1.PDF]

Supplementary Table 1. Overview over median relative abundances and interquartile range, as well as over significant changes of the 28 most abundant genera.

Genus		Timepoint 1	Timepoint 2	Timepoint 3	Timepoint 4	Timepoint 5	Timepoint 6
Bacteroides	Median	15.63	72.87	83.46	14.98	12.92	4.49
	IR[1]	2.87-41.52	1.00-85.63	60.06-89.77	6.21-50.57	3.47-19.13	3.43-26.24
	P[2]	-	-	-	-	-	-
Escherichia-Shigella	Median	0.77	0.93	0.17	2.25	0.00	0.09
	IR[1]	0.42-91.19	0.01-21.99	0.02-0.74	0.80-18.50	0.00-0.01	0.00-0.60
	P[2]	5, 6	6	-	-	1	1, 2
Faecalibacterium	Median	5.47	2.72	3.57	0.61	0.14	0.49
	IR[1]	2.4411.98	1.39-15.15	2.59-9.33	0.41-4.64	0.05-0.25	0.35-2.13
	P[2]	5, 6	6	6	5	1, 4	1, 2, 3
Paraprevotella	Median	0.03	0.01	0.01	11.62	30.49	3.53
	IR[1]	0.00-11.00	0.00-0.02	0.00-3.94	0.68-49.38	23.18-41.08	1.64-6.38
	P[2]	5, 6	-	-	5	1, 4	1
Sutterella	Median	0.00	0.01	2.80	0.41	0.77	0.01
	IR[1]	0.00-0.01	0.00-2.51	0.00-17.13	0.12-4.96	0.09-3.12	0.00-0.50
	P[2]	3, 4, 5	-	1	1	1	-
Rikenella	Median	0.36	0.00	0.02	2.79	2.70	11.22
	IR[1]	0.00-4.12	0.00-0.04	0.00-3.70	0.92-33.20	0.81-11.14	1.88-27.15
	P[2]	-	-	-	-	-	-
Butyricicoccus	Median	0.15	1.92	0.18	0.29	1.46	2.51
	IR[1]	0.05-1.72	0.05-17.53	0.11-0.84	0.09-7.59	0.68-3.36	0.56-4.85
	P[2]	2	1	-	-	-	-
Parabacteroides	Median	0.07	0.07	1.01	0.68	0.24	0.05
	IR[1]	0.00-0.43	0.01-13.58	0.21-1.61	0.33-2.09	0.12-0.53	0.00-0.35
	P[2]	-	-	-	-	-	-

[1]IR = Interquartile range (25 to 75%)

[2]*P* = significant difference (P<0.05) to the timepoint given

Supplementary Table 1. Continued.

Genus		Timepoint 1	Timepoint 2	Timepoint 3	Timepoint 4	Timepoint 5	Timepoint 6
Lactobacillus	Median	0.38	0.14	0.29	0.00	0.00	0.00
	IR[1]	0.06-1.38	0.04-4.96	0.06-0.87	0.00-2.94	0.00-0.06	0.00-0.01
	P[2]	5, 6	6	6	-	1	1, 2, 3
Oscillibacter	Median	0.35	0.01	0.46	0.92	7.01	12.72
	IR[1]	0.02-2.47	0.00-0.17	0.01-1.52	0.12-5.39	4.04-9.10	3.50-26.09
	P[2]	-	-	-	-	6	5
Paludibacter	Median	0.09	0.00	0.00	0.33	4.39	0.57
	IR[1]	0.00-1.52	0.00-0.02	0.00-0.07	0.05-13.44	1.50-10.72	0.06-9.55
	P[2]	-	-	-	-	-	-
Pseudomonas	Median	0.40	0.00	0.00	0.00	0.00	0.00
	IR[1]	0.00-19.35	0.00-0.01	0.00-0.00	0.00-0.00	0.00-0.02	0.00-0.02
	P[2]	-	-	-	-	-	-
Phocaeicola	Median	0.13	0.00	0.00	0.00	0.12	10.35
	IR[1]	0.00-1.77	0.00-0.00	0.00-0.1	0.00-0.07	0.00-12.97	4.47-17.71
	P[2]	-	6	6	6	-	2, 3, 4
Lachnospiraceae incertae sedis	Median	0.66	0.01	0.02	0.39	1.00	1.49
	IR[1]	0.05-2.65	0.00-1.13	0.00-0.06	0.04-0.52	0.51-1.90	0.61-1.87
	P[2]	2	1	-	-	-	-
Alistipes	Median	0.07	0.00	0.02	0.00	0.24	2.44
	IR[1]	0.01-0.97	0.00-0.00	0.00-0.27	0.00-0.22	0.11-2.20	0.35-6.80
	P[2]	2	1	-	6	6	4, 5
Clostridium XIVa	Median	0.22	0.29	0.19	0.08	0.24	0.16
	IR[1]	0.12-0.30	0.21-1.13	0.05-0.75	0.07-0.18	0.14-0.50	0.05-0.22
	P[2]	6	6	-	-	-	1, 2

[1]IR = Interquartile range (25 to 75%)

[2]*P* = significant difference ($P<0.05$) to the timepoint given

Supplementary Table 1. Continued.

Genus		Timepoint 1	Timepoint 2	Timepoint 3	Timepoint 4	Timepoint 5	Timepoint 6
Clostridium XIVb	Median	0.03	0.00	0.02	0.24	0.18	0.15
	IR[1]	0.00-0.69	0.00-0.78	0.00-0.06	0.04-0.38	0.08-0.44	0.01-0.38
	P[2]	-	-	-	-	-	-
Klebsiella	Median	0.00	0.00	0.00	0.00	0.00	0.00
	IR[1]	0.00-0.52	0.00-0.00	0.00-0.01	0.00-1.88	0.00-0.01	0.00-0.02
	P[2]	-	-	-	-	-	-
Flavonifractor	Median	0.07	0.04	0.31	0.43	0.18	0.09
	IR[1]	0.00-0.16	0.00-0.29	0.11-1.21	0.15-1.48	0.07-0.40	0.04-0.23
	P[2]	-	-	4	3	-	-
Sporobacter	Median	0.00	0.00	0.00	0.08	0.32	0.69
	IR[1]	0.00-1.39	0.00-0.00	0.00-0.01	0.02-0.53	0.25-1.15	0.18-1.52
	P[2]	-	-	-	-	-	-
Butyricimonas	Median	0.00	0.00	0.00	0.00	0.19	0.08
	IR[1]	0.00-0.00	0.00-0.00	0.00-0.01	0.00-0.02	0.05-0.55	0.00-0.81
	P[2]	-	-	-	-	-	-
Ahrensia	Median	0.00	0.00.	0.17	0.04	0.00	0.00
	IR[1]	0.00-0.00	0.00-0.00	0.00-1.84	0.00-0.25	0.00-0.02	0.00-0.09
	P[2]	-	-	-	-	-	-
Odoribacter	Median	0.03	0.00	0.01	1.35	0.80	0.01
	IR[1]	0.00-0.16	0.00-0.01	0.00-0.48	0.03-1.88	0.20-2.69	0.00-0.28
	P[2]	-	-	-	-	6	5
Acinetobacter	Median	0.19	0.00	0.00	0.00	0.00	0.00
	IR[1]	0.00-1.44	0.00-0.00	0.00-0.00	0.00-0.00	0.00-0.00	0.00-0.00
	P[2]	-	-	-	-	-	-

[1]IR = Interquartile range (25 to 75%)

[2]*P* = significant difference ($P<0.05$) to the timepoint given

Supplementary Table 1. Continued.

Genus		Timepoint 1	Timepoint 2	Timepoint 3	Timepoint 4	Timepoint 5	Timepoint 6
Barnesiella	Median	0.00	0.00	0.00	0.00	0.93	0.70
	IR[1]	0.00-0.08	0.00-0.00	0.00-0.03	0.00-0.44	0.30-1.67	0.01-2.99
	P[2]	6	-	-	-	-	1
Ethanoligenens	Median	0.00	0.00	0.00	0.00	0.33	0.19
	IR[1]	0.00-0.18	0.00-0.00	0.00-0.01	0.00-1.88	0.00-0.01	0.00-0.02
	P[2]	-	-	-	-	-	-
Butyrivibrio	Median	0.00	0.00	0.00	0.00	0.28	0.26
	IR[1]	0.00-0.07	0.00-0.00	0.00-0.01	0.00-1.27	0.09-1.92	0.07-1.11
	P[2]	-	-	-	-	-	-
Clostridium IV	Median	0.08	0.00	0.03	0.05	0.29	0.81
	IR[1]	0.00-0.50	0.00-0.02	0.01-0.15	0.02-0.17	0.12-0.57	0.22-2.20
	P[2]	-	-	-	-	-	-

[1]IR = Interquartile range (25 to 75%)

[2]*P* = significant difference ($P<0.05$) to the timepoint given
